# Proceedings of the American Medico-Psychological Association at the Fifty-Fourth Annual Meeting, Held at St. Louis, Mo., May 10-13, 1898

**Published:** 1899-09

**Authors:** 


Proceedings of the American Medico-Psychological Association at the Fifty-fourth annual meeting, held at St. Louis, Mo., May 10-13, 1898. Published by the Association. Edited by the Secretary, Dr. C. B. Burr, Flint, Michigan.

This volume contains some twenty-five papers on subjects interesting to the alienist. The presidential address, delivered by Dr. Bucke, of London, Ont., clearly points out that many cures result among the female insane from operations upon the reproductive organs when they are diseased. Dr. Eskridge, in the annual address, outlines very completely the requirements of a hospital for the study of scientific psycho-neurology. Dr. Brower's excellent essay on the judicious training of neurotic children is sufficiently clear and concise to be read with advantage by the laity as well as members of the profession. The other contributions are well written and ably discussed. The publisher's work is also well done. J. W. P.

